# Life on the fringe: microbial adaptation to growth on carbon monoxide

**DOI:** 10.12688/f1000research.16059.1

**Published:** 2018-12-27

**Authors:** Frank T. Robb, Stephen M. Techtmann

**Affiliations:** 1 Department of Microbiology and Immunology, and Inst of Marine and Environmental Technology, University of Maryland, Baltimore, Baltimore, MD, 21202, USA; 2 Department of Biological Sciences, Michigan Technological University, 1400 Townsend Drive, Houghton, MI, 49931, USA

**Keywords:** carbon monoxide, CO dehydrogenase, extremophile

## Abstract

Microbial adaptation to extreme conditions takes many forms, including specialized metabolism which may be crucial to survival in adverse conditions. Here, we analyze the diversity and environmental importance of systems allowing microbial carbon monoxide (CO) metabolism. CO is a toxic gas that can poison most organisms because of its tight binding to metalloproteins. Microbial CO uptake was first noted by Kluyver and Schnellen in 1947, and since then many microbes using CO via oxidation have emerged. Many strains use molecular oxygen as the electron acceptor for aerobic oxidation of CO using Mo-containing CO oxidoreductase enzymes named CO dehydrogenase. Anaerobic carboxydotrophs oxidize CO using CooS enzymes that contain Ni/Fe catalytic centers and are unrelated to CO dehydrogenase. Though rare on Earth in free form, CO is an important intermediate compound in anaerobic carbon cycling, as it can be coupled to acetogenesis, methanogenesis, hydrogenogenesis, and metal reduction. Many microbial species—both bacteria and archaea—have been shown to use CO to conserve energy or fix cell carbon or both. Microbial CO formation is also very common. Carboxydotrophs thus glean energy and fix carbon from a “metabolic leftover” that is not consumed by, and is toxic to, most microorganisms. Surprisingly, many species are able to thrive under culture headspaces sometimes exceeding 1 atmosphere of CO. It appears that carboxydotrophs are adapted to provide a metabolic “currency exchange” system in microbial communities in which CO arising either abiotically or biogenically is converted to CO _2_ and H _2_ that feed major metabolic pathways for energy conservation or carbon fixation. Solventogenic CO metabolism has been exploited to construct very large gas fermentation plants converting CO-rich industrial flue emissions into biofuels and chemical feedstocks, creating renewable energy while mitigating global warming. The use of thermostable CO dehydrogenase enzymes to construct sensitive CO gas sensors is also in progress.

## Introduction

**Figure 1.**
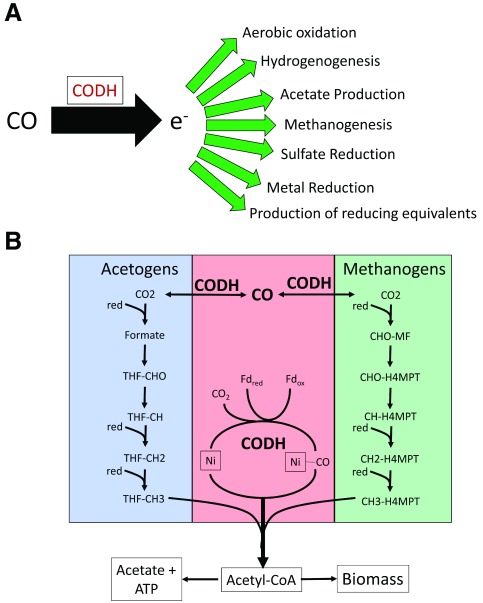
Carbon monoxide (CO) metabolisms and the Wood–Ljungdahl pathway. ( **A**) Potential fates of CO in microbial physiologies. ( **B**) Wood–Ljungdahl pathway. The carbonyl pathway is shown in pink, and the role of the CODH complex is highlighted. The generalized methyl branch of the pathway used in acetogenic bacteria is shown in blue. The generalized methyl branch of the pathway used in methanogenic archaea is shown in green.

Public perception of the role of carbon monoxide (CO) in biology is dominated by its reputation as a silent killer because of its toxicity. Toxicity results from tight binding of CO to the metallocenters in heme proteins, such as hemoglobin, myoglobin, and cytochrome oxidase ^1^. Globally, CO is considered an atmospheric trace gas and rarely exceeds 1 ppm except in heavily polluted city airspaces, volcanic exhalations, or industrial flue gases ^2^. Volcanic exhalations have significant CO content, submarine hydrothermal vent fluids have about 100 nM CO, and local pockets of moderate concentrations of CO are produced biogenically by bacterial fermentation ^3^ or in soil associated with rhizosphere bacteria ^4, 5^. CO has high potential as an electron donor (E −524 to 558 mV for the CO/CO _2_ couple) ^6^. Therefore, apart from its toxicity, CO represents a very favorable energy and carbon source for microbial growth. In this short review, we will address aspects of microbial metabolism allowing CO utilization through CO oxidoreductase enzymes. Historically, evidence for CO utilization by “methane bacteria” was noted by Kluyver and Schnellen in 1947 ^7^ and by Schlegel ^8^. The pathway for energy generation in phototrophic bacteria whilst growing anaerobically in the dark ^9^ is now known to be a widespread metabolic capability in many species of bacteria and archaea as well as some fungi and algae. CO forms a remarkable metabolic network with many pathways (as shown in Figure 1A), including globally significant carbon cycling routes via acetogenesis and methanogenesis, which both involve anaerobic pathways in which CO is a key intermediate ^10^. The Wood–Ljungdahl (WL) pathway depicted in Figure 1B is a highly adaptable set of enzymes that allow acetate formation by acetogens as well as anaplerotic feeding through the production of acetyl-CoA in many autotrophic bacteria and archaea. Recently, microbial isolates and consortia that carry out hydrogenogenic carboxydotrophy have been identified and shown to be common in many environments. These organisms link energy-conserving hydrogenases with CO dehydrogenases.

## The organisms: CO as the source of energy and fixed carbon

Early studies by Kistner yielded an aerobic bacterial isolate from sewage sludge that could oxidize CO ^11^, and this physiology is now represented by a large and diverse group of aerobic CO-oxidizing bacteria that use molecular O _2_ as an electron acceptor, reviewed by King and Weber ^4^. Carboxydotrophs are a diverse and eclectic set of organisms ranging from the Proteobacteria to the Firmicutes and some archaea. Anaerobic CO oxidation in purple bacteria was described by Uffen ^9^ in the 1970s and Kistner ^11^ in the 1950s, leading to steady advances in the microbiology of CO metabolism. A large variety of coupled metabolic processes and synergistic microbial metabolic interactions have now been discovered. ****

The substantial number of emerging bacterial and archaeal genomes containing CODH catalytic subunit encoding genes ^9^ reveal that many classes of bacteria use CO oxidation either as a standby energy conservation strategy or as their major carbon source. Table 1 shows a selection from these strains together with their major CO-utilization pathways ^9– 21^.

**Table 1.** Representative carboxydotrophic microbial species and their physiological features.

CO-utilizing species	Optimal temperature, °C	CO as energy source	CO as carbon source	Reference	CO pathways
*Rubrivivax gelatinosus*	34	+	−	12	Ech
*Rhodospirillum rubrum*	30	+	−	28	Ech
*Citrobacter sp Y19*	35	+	+	13	Ech
*Carboxydothermus hydrogenoformans* *Z2901*	72	+	+	10	Ech, WL, R
*Thermosinus carboxydivorans*	60	+	+	17	Ech, WL
*Carboxydocella thermautotrophica*	58	+	+	16	Ech, WL
*Caldanaerobacter subterraneus subsp.* *pacificus*	70	+	?	15	Ech, WL
*Thermococcus AM4*	82	+	−	18	Ech
*Thermococcus onnurineus NA1*	85	+	−	19	Ech
*Methanobacterium* *thermoautotrophicum*	55	+	+	19	WL ^a^
*Oligotropha carboxidovorans OM5*	30	+	−	29	Ox
*Thermogemmatispora carboxidivorans* *PM5*	55	+	−	21	Ox
*Thermomicrobium roseum*	55	+	−	22	Ox

CO, carbon monoxide; Ech, energy-conserving hydrogenase; Ox, oxygen as electron acceptor; R, redox reactive; WL, Wood–Ljungdahl. ^a^This methanogen strain can grow on CO, producing methane.

CO can fuel a number of metabolisms, including aerobic carboxydotrophy, acetogenesis, methanogenesis, and sulfate reduction. Many carboxydotrophic strains are capable of oxidizing CO with water to form CO _2_ and H _2_, known as the water–gas shift reaction, as follows: CO + H _2_O ⇔ CO _2_ + H _2_.

This physiology has been well studied in isolates capable of performing hydrogenogenic carboxydotrophy, found in anaerobic bacteria and archaea ^22^. A number of these thermophilic hydrogenogens are found within the genera *Carboxydothermus*, *Thermincola*, and *Carboxydocella*. Some other isolates such as *Desulfotomaculum carboxydivorans* can couple CO oxidation to sulfate reduction. Other isolates such as *Carboxydocella thermautotrophica* ^23^, *Thermosinus carboxydivorans* ^24^, and *Carboxydothermus ferrireducens* ^17^ are able to link CO oxidation to metal reduction in addition to sulfate reduction. While both methanogens and acetogens employ CO as an intermediate in the WL pathway and use CO as a direct input into the pathway, a subset of these organisms can also produce CO directly ^25^. Some methanogens, including *Methanosarcina acetivorans*, *Methanosarcina barkeri*, and *Methanothermobacter thermautotrophicus*, can grow on CO with methane production. *M. acetivorans* has a very versatile metabolic response, being able to grow on CO and produce acetate and formate as end products, rather than methane ^26^. Acetogens such as *Moorella thermoacetica* can use CO as an input into the WL pathway for autotrophic growth and acetate production ^20, 27^. As genomic and metagenomic studies are increasingly used to inform physiology, it is likely that many more strains able to use CO will be identified.

## Key enzymes: aerobic and anaerobic CO dehydrogenases and acetyl-CoA synthases

Oxidation of CO to CO _2_ is an exergonic reaction catalyzed in either anaerobic conditions (using many different electron acceptors) or aerobically (using molecular oxygen as the electron acceptor). Two fundamentally distinct classes of CO dehydrogenase catalyze globally significant metabolic reactions that result in CO transformation ^4, 27^. In anaerobes, the CODH active site contains sulfur-coordinated Ni in a cubane [Ni–4Fe–5S] center called the C-cluster ^30, 31^, whereas aerobes have heterotrimeric enzymes named CO dehydrogenase that belong to the xanthine oxidase family with an active site containing a binuclear cluster of Mo and Cu (MoCu–CODH) ^28, 29^. The CO dehydrogenase enzyme is a dimer of two heterotrimers, each composed of the coxL subunit (molybdenum protein), a flavoprotein, and an iron–sulfur protein (FeSP). The CO dehydrogenase enzyme complex is monofunctional, resulting in rapid unidirectional conversion of CO to CO _2_. In contrast, the anaerobic enzymes have a Ni catalytic site and are known as CODH. They are encoded by the *CooS* gene and catalyze the reversible conversion of CO and H _2_O to CO _2_. A FeSP serves as both electron acceptor and donor in the reaction—CO + H _2_O + FeSP ⇌ CO _2_ + FeSP ^2−^ + 2H ^+^—where both the forward and the backward reactions are very important, as they are central to anaerobic metabolic pathways of great biological significance. The CODH enzyme is found in many anaerobic and facultative microorganisms, both bacteria and archaea, but is absent in aerobic microorganisms. The coordination of nickel in the active site of all nickel-containing CO dehydrogenases appears to be very similar to that first revealed by Dobbek *et al*. ^31^. Some anaerobic CODHs are monofunctional, producing CO _2_; others are bifunctional and form complexes with tightly bound accessory subunits, producing products including acetyl-CoA and H _2_^31^.

Table 2 lists the multiple CO-linked pathways that have been described; in these pathways, CO oxidation results in many alternative end products, including hydrogen, acetate, formate, reduced metal species, ethanol, and butanol.

**Table 2.** Coupled reactions resulting from the activities of CODH enzymes, with the molar free energy of reaction.

Product	Reaction Reactions from CO	ΔG ^0^ ^a^, kJ mol CO ^−1^
Formate	CO + H _2_O à HCOO ^−^ + H ^+^	−16
Hydrogen	CO + H _2_O à H _2_ + CO _2_	−20
Ethanol	6CO + 3H _2_O à CH _3_CH _2_OH + 4CO _2_	−37
n-Butanol	12CO + 5H _2_O à CH _3_(CH _2_) _3_OH + 8CO _2_	−40
Acetate	4CO + 2H _2_O à CH _3_COO ^−^ + H ^+^ + 2CO _2_	−44
Butyrate	10CO + 4H _2_ à CH _3_(CH _2_)2COO ^−^ + H ^+^ + 6CO _2_	−44
Methane	4CO + 2H _2_O à CH _4_ + 3CO _2_	−53
	**Reactions from H _2_/CO**	
Methanol	CO + 2H _2_ à CH _3_OH	−39
Acetate	2CO + 2H _2_ à CH _3_COO ^−^ + H ^+^	−67
Ethanol	2CO + 4H _2_ à CH _3_CH _2_OH + H _2_O	−72
Butyrate	4CO + 6H _2_ à CH _3_(CH _2_) _2_COO ^−^ + H ^+^ + 2H _2_O	−80
n-Butanol	4CO + 8H _2_ à CH _3_(CH _2_) _3_OH + 3H _2_O	−81
Methane	CO + 3H _2_ à CH _4_ + H _2_O	−151

^a^ΔG ^0^ values at standard temperature and pressure.

The free energy of reaction for these exergonic couples is shown. The large ΔG ^0^ (151 kJ mole CO) resulting from methanogenesis from CO _2_ and H _2_ is notable. The bifunctional CODH responsible for this reaction has been characterized as part of the carbonyl branch of the WL pathway ^32^ ( Figure 1). Another key enzyme in WL pathways is the acetyl-CoA synthase (ACS), which catalyzes the reaction of CO and CH _3_ to produce acetyl-CoA ^32^. CO can be generated *in situ* from CO _2_ via the CODH and channeled to the ACS complex through direct interaction of the CODH and ACS via a gas tunnel inside the complex or directly input into the ACS in systems where CO is present ^33^. The ACS complex is reversible, and in acetoclastic methanogens, the process can run in reverse to split the acetyl-CoA into CoA, methylated tetrahydrosarcinapterin (CH3-M4MPT), and CO ^33, 34^. The WL pathway is a major contributor to carbon cycling in anaerobic environments and contributes to global carbon fixation along with the Calvin cycle and the reductive tricarboxylic acid cycle pathway. The WL pathway is the most ancient of the three ^35^, although the other two pathways are more prevalent on modern Earth.

Phylogenetic analysis of the catalytic subunits of the monofunctional and bifunctional enzymes has indicated that the enzyme phylogeny does not follow taxonomic distinctions ^35^. There are a number of cases where non-related organisms have very similar catalytic subunits. Thorough analysis of genomes of carboxydotrophs has established that the monofunctional CODH can be horizontally transferred, leading to a widely distributed gene cluster in both the bacteria and the archaea ^12^. A study performed in 2012 found that 6% of bacterial and archaeal genomes contain genes for either a monofunctional or a bifunctional anaerobic CODH gene cluster, suggesting that these genes are widely distributed throughout the tree of life through horizontal gene transfer ^12^. In a more recent study focused exclusively on the bifunctional CODH, this cluster was found in 143 bacterial genomes and 106 archaeal genomes ^12^. This suggests that the CODH gene cluster has been disseminated by lateral gene transfer across broad taxonomic divides. These gene clusters have been found in many of the recently described candidate phyla identified through metagenomic assembled genomes (MAGs) and representing archaea that resist cultivation ^35^. The “virtual” archaeal phyla, Thorarchaeaota and Lokiarchaeota, which are of particular interest as they have been proposed as close relatives of the eukaryotic ancestor, both contain CODH/ACS clusters ^36^.

The presence of the CODH gene cluster in both bacteria and archaea has been used to suggest an ancient origin of CODH. Recently, the WL pathway was proposed as the ancestral carbon fixation pathway in the last universal common ancestor (LUCA) ^37^. However, it is important to note that the WL pathway can operate both catabolically and anabolically and current data do not allow us to conclude that the CODH/ACS in LUCA was of the anabolic variety. CODH has been coupled to a variety of other metabolic processes in addition to the CODH/ACS complexes. The CODHs in hydrogenogenic carboxydotrophs have the ability to interact with a membrane-bound energy-conserving hydrogenase that is capable of generating hydrogen and extracellular protons ^35^. Other coupled systems have linked CO oxidation to the production of reducing equivalents by driving the reduction of NAD (P) to NAD (P)H, coping with oxidative stress ^38^, and providing electrons for metal reduction ^14^. Many of these biochemical linkages are also reflected in the genetic proximity of *cooS* to the functional genes in clusters that enable coregulation of the coupled functions and thus guides to the annotation of the coordinated metabolic pathways. However, there are many cases in which the *cooS* gene has been found in the genome unaccompanied by annotated functional genes for CO metabolism ^14, 17^. The function of many lone *cooS* genes remains unknown ^12^.

Genomes often encode more than one CODH ^12^. The multiplicity of CODHs within the genome in many species hints at the diversity of metabolic processes that could be linked to CO. CODH homologs must couple to their functional partners through exclusive protein–protein interactions, thus requiring different catalytic subunits for each functional pathway in the cell. The binding surfaces must evolve to assemble into enzyme complexes in orderly folding pathways. The CODH–ACS complexes have evolved remarkable structures with precision joining of tunnels for conduction of reactant molecules between the active sites ^12^, thus maintaining the rapid turnover of substrates and cofactors. The versatile carboxydotroph *Carboxydothermus hydrogenoformans* encodes five CODH gene clusters ^39^, and although this record number of CODH gene clusters was thought to be an anomaly, recent genomic work has indicated several other organisms that encode equal numbers of paralogs of CODH genes. *C. hydrogenoformans* has the ability to regulate the distribution of CO into different pathways by gene regulation through two different CO-binding CooA proteins that bind CO and induce CO gene clusters differentially at different CO concentrations ^14^.

Another aspect of CODH function is the maturation of the enzyme, which involves the correct insertion of Ni and Fe into the active site ^40^. Although it is clear that CODH depends on the accessory protein CooC (a 30 kDa ATPase) for maturation, the mechanisms and accessory proteins/chaperones involved in the maturation of C-cluster are emerging from recent studies ^41^. These studies suggest that the maturation of the active site cubane Ni cluster may differ from one enzyme to another. In most cases, the maturation of CODH in recombinant production systems is dependent on the specific maturase activity of *cooC* gene product and the proteins must be co-expressed in order to produce CODH into which Ni is fully delivered ^42^. Mutations in the CooC protein that cripple ATPase activity cause a deficiency in CO-dependent growth that is reversed only when high concentrations of Ni are added to the culture medium to compensate for the deficient maturation pathway ^43^.

## The environment: distribution of CO-related genes in the environment

Carboxydotrophs can be found in most thermal environments ^44^ and in some non-thermal terrestrial habitats ^45^. Whereas the initial characterization of CO utilization was in mesophilic organisms, subsequent isolations, particularly from thermal environments, have uncovered diverse carboxydotrophic microbes. The concentrations of CO in volcanic exhalations range from 50 to 110 ppm in Italy ^4^; however, it is likely that biogenic CO in dense microbial consortia (that is, hot spring or hydrothermal vent mats) is much higher, as sulfate-reducing bacteria can accumulate up to 600 ppm in culture during late stationary phase ^46^. Significantly, CO in the Earth’s atmosphere, though between 50 and 160 ppb, is increasing regionally in the areas with large-scale anthropogenic inputs ^3^.

In addition to these cultured members, molecular analysis of thermal environments has indicated that carboxydotrophic metabolisms and CO-related genes are commonplace in these thermal settings. One recent study used stable isotope probing to inventory the anaerobic carboxydotrophs in geothermal springs ^2^. The authors found that relatives of *Thermincola*, *Desulfotomaculum*, *Thermolithobacter*, and *Carboxydocella* were commonly co-labeled using ^13^C isotopically labeled CO. This finding suggests that the ability to use CO is common in hot springs and many of the relatives of isolated carboxydotrophs are active players in these geothermal settings. Another recent study demonstrated that, in some hot springs, CO was actively consumed under anaerobic conditions during incubation, suggesting an active carboxydotroph community ^47^. Additionally, the abundance of a specific *cooS* gene was quantified by using quantitative polymerase chain reaction. This work demonstrated that this *cooS* gene was present in some hot springs at greater than 10 ^4^ copies per gram of sediment. Although the copies of bacterial and archaeal 16S rRNA genes were often two orders of magnitude more abundant than the *cooS* gene, in some hot springs the *cooS* gene abundance was the same as the 16S rRNA gene abundance. These findings combine to suggest that in volcanic thermal settings CO is actively consumed by an abundant group of anaerobic carboxydotrophs.

Although the presence of CO-based metabolism is widely accepted in hot spring and deep-sea volcanic vent settings, the abundance of carboxydotrophs and CO-related genes in non-thermal locations is under-appreciated. Recent work has indicated that CO-associated genes are common in deep-sea sediments ^48^. This could be due in part to the presence of CO-related genes in members of these uncultivated phyla present in the deep ocean ^49^. Another recent study detected the presence of *CooS* genes throughout surface sediment in the oceans as well as in subsurface sediments down to 390 m ^36^.

Rock-hosted subsurface ecosystems are also heavily influenced by microbes that encode CO-related genes. Environments influenced by serpentinization, the aqueous alteration of iron-rich mantle rocks, commonly generate hyperalkaline conditions with copious reduced gas, including CO. Metagenomic analysis of serpentinite-hosted ecosystems has indicated the presence of CO-related genes ^49, 50^. Because these systems are limited in dissolved inorganic carbon, it is suggested that these organisms are able to use CO as both an electron donor and a carbon source ^51^.

However, the presence of carboxydotrophs and CO-related genes is not limited to conventional extreme environments. Oddly, genes involved in the CODH/ACS pathway in serpentinite systems have close homologs in metagenomes of the microbiome of the termite hindgut ^51^. Like serpentinite habitats, the termite hindgut is an extremely alkaline milieu. Many of the recovered *CooS* genes were implicated in homoacetogenic metabolism ^52^.

These findings combine to suggest that microbial CO metabolism is more widespread than previously thought. However, many of the locales in which carboxydotrophs have been found have sparingly low concentrations of CO. In some settings where CO is produced through abiotic processes, the role of carboxydotrophic microbes may be to provide a strategy for detoxification. Carboxydotrophs can remove CO, which is toxic to many consortium members at high concentrations, and produce CO _2_ and H _2_, gases which cross-feed to other pathways such as methanogenesis and sulfate reduction ^53^. Therefore, an alternative model is one in which carboxydotrophs act in a form of currency exchange between organisms that can produce CO and CO-utilizing organisms. There is strong evidence that sulfate reducers and methanogens can produce CO as an end product of their metabolism, similar to fermentation end products in heterotrophic microbes. For example, as mentioned previously, the sulfate reducer *Desulfovibrio vulgaris* produces high levels of CO and certain mutants produce up to 600 ppm of CO during stationary phase ^3^. Some methanogens produce CO during growth ^3^. In mat or biofilm communities, locally high levels of CO may accumulate while the bulk CO levels remain low. Our conceptual models would suggest that biogenic CO can be used by carboxydotrophic microbes to fuel their metabolism and produce compounds such as H _2_, CO _2_, or acetate which are able to fuel other metabolisms such as methanogenesis and sulfate reduction. This form of inter-species currency exchange via CO or detoxification of CO suggests a central role for carboxydotrophs in many anaerobic ecosystems.

## Biotechnology and industrial applications

Adapting carboxydotrophs to create novel and interesting large-scale processes has been increasing rapidly. Discoveries that CO can be not only used to generate H _2_ but also converted directly into a variety of liquid fuel components and chemical feedstocks, including ethanol, N-butanol, and acetone, have sparked a new field of research. There are several pilot-scale projects under way using large fermenters sparged with industrial flue gases, responding to incentives related to reduced carbon emissions. Some are coupled to hydrogen production; however, the acetogenic gas fermentation can be adapted to produce solvents from syngas or refinery flue gases in many geographic regions without competing for food or land. The production of ethanol or butanol ^26^ and other chemical feedstocks by pilot installations using carboxydotrophs is now quite well established ^54^. The application of metabolic engineering to adapt the mesophilic acetogen *Clostridium autoethanogenum* ^55^ to large-scale gas fermentation using a range of industrial effluent gas streams is becoming sophisticated and production is now coming on stream ^56^.

Another aspect is the development of gas sensors based on immobilized thermostable enzymes ^55^. Biosensors with CO-inert electrodes combined with immobilized CO oxidoreductase from the thermophile *Pseudomonas thermocarboxovorans* using phenazine ethosulfate as the electron acceptor (with a K _m_ of 3.8 µM) have shown potential for CO detection within the ppm to ppb range with durability and rapid response ^57^.

## Conclusions and future directions

CO-based metabolism is surprisingly common in extremophilic and non-extremophilic organisms and is widely distributed throughout the tree of life. Furthermore, there is good evidence that the WL pathway is the most ancient carbon fixation route, making CO-based metabolism an ancestral physiology ^58^. Diversification of the pathway during evolution allowed CO oxidation to be coupled to various metabolic processes ranging from energy conservation to carbon acquisition, metal reduction/detoxification, and coping with oxidative stress. As burgeoning metagenomic data have revolutionized our understanding of microbial ecology, CO-related genes have been commonly found in MAGs throughout the world. This widely distributed metabolism also has great potential for recently invented industrial applications aimed at alleviating carbon emissions and mitigating climate change.

There is still much to be clarified regarding the mechanism of CO-based metabolisms. Although both monofunctional and bifunctional CODHs have been found scattered throughout the tree of life, the mechanisms of metabolic coupling to CODHs are still partially explored and the electron transfer coupling routes are in many cases not clear. Genomic synteny often underscores potential linkages; however, more biochemical characterization of monofunctional CODHs is required in order to better predict the function of CODH from genomic data, especially for CODHs that are found in MAGs or single amplified genomes (SAGs) from uncultivated microbes. However, biotechnology innovation is now being vigorously applied to use CO as a feedstock in novel large-scale bioengineering applications in major carbon-emitting countries, potentially lowering the carbon footprint of some heavy industries like steel production and potentially curbing the relentless trend of global warming.
